# Associations Among Online Health Information Seeking Behavior, Online Health Information Perception, and Health Service Utilization: Cross-Sectional Study

**DOI:** 10.2196/66683

**Published:** 2025-03-14

**Authors:** Hongmin Li, Dongxu Li, Min Zhai, Li Lin, ZhiHeng Cao

**Affiliations:** 1 School of Public Health Jining Medical University Jining China; 2 Rencheng District Center for Disease Control and Prevention Jining China

**Keywords:** online health information seeking (OHIS), online health information perception (OHIP), mediating effect, health service utilization, health information, health perception, data, China, Chinese General Social Survey (CGSS), database, medical information, survey

## Abstract

**Background:**

Seeking online health information can empower individuals to better understand their health concerns, facilitating their ability to manage their health conditions more effectively. It has the potential to change the likelihood and frequency of health service usage. Although existing literature has demonstrated the prevalence of seeking online health information among different populations, the factors affecting online health information perception and discussions on the associations between seeking online health information and health service utilization are limited.

**Objective:**

We analyzed the associations between online health information seeking behavior and health service utilization, as well as the online health information perception delivery mechanism.

**Methods:**

We analyzed data from the Chinese General Social Survey, the first national representative survey conducted in mainland China. The independent variable was the online health information seeking behavior. The outcome variable was health service utilization by the respondents, and online health information perception was selected as the mediating variable in this analysis. Factor analysis was conducted to obtain online health information perception. Multiple regressions were performed to investigate the effect of online health information seeking behavior on physician visits. Bootstrap methods were conducted to test the mediation effects of online health information perception.

**Results:**

This analysis included 1475 cases. Among the participants, 939 (63.66%) sought online health information in the last 12 months. The mean age of the respondents was 46.72 (SD 15.86) years, and 794 (53.83%) were females. After controlling for other variables, individuals with online health information seeking behaviors showed 0.289 times more outpatient visits (*P*=.003), 0.131 times more traditional Chinese medicine outpatient visits (*P*=.01), and 0.158 times more Western medicine outpatient visits (*P*=.007) over the past year compared to those who did not seek health information online. Additionally, multiple regression analyses revealed statistically significant effects of gender, age, and health status on physician visits. The total effect revealed that seeking online health information significantly influenced the total physician visits (β=0.290; *P*=.003), indicating a certain correlation between online health information seeking behavior and physician visits. Seeking online health information had a significant positive impact on the perception (β=0.265; *P*<.001). The mediation effects analysis identified that online health information perception led to a significant increase in physician visits with the increase in the online health information seeking behaviors (β=0.232; *P*=.02).

**Conclusions:**

The online health information perception of an individual influences the effect online health information seeking has on the frequency of physician visits. The online health information seeking behavior impacts outpatient service utilization both directly and indirectly through online health information perception and significantly increases the frequency of clinic visits after controlling for other variables. Interventions can be explored to improve the health utilization of residents by increasing their online health information perception.

## Introduction

The internet has become an essential part of life around the world. In 2023, the number of internet users reached 5.3 billion globally. Internet penetration rates in Saudi Arabia, Norway, and the United Arab Emirates are approaching 100% [1]. In China, which is the leading market for internet users, the online penetration rate was 78% in June 2024, with 1.099 billion users [2]. There is no doubt that the internet has become the most common resource for health information due to the widespread use of smartphones. Our previous study found that even in rural and remote areas in China, 47.27% of the residents obtained health-related information or services through the internet [3].

Individuals can easily access internet effectively, obtain the relevant medical information available online, and make health care decisions based on their health status. Online health information seeking (OHIS) can empower individuals to better understand their health concerns, facilitating them to manage their health conditions more effectively. Recent studies on OHIS behavior have focused on the population characteristics and associated factors. OHIS behavior in different populations [4] have been discussed, such as college students [5,6], older adults [7,8], adolescents [9,10], mothers [11], and people with various health statuses [12]. OHIS behavior and its influencing factors have also been analyzed in patients with specific diseases such as cancer [13-15], osteoarthrosis [16], type 2 diabetes mellitus [17,18], and rare diseases [19]. The OHIS behavior is influenced by various factors, including demographics, psychological aspects, role-related or interpersonal traits, environmental sources, source-related characteristics, and diseases [13-19]. OHIS can enable individuals to assess their health concerns better, facilitating them to improve their management of health conditions and make more informed decisions about whether to use health services. Therefore, OHIS can potentially change the likelihood and the frequency of health service usage, such as outpatient visits. A systematic review suggested that the current evidence of the association between OHIS and health services is inconclusive [20]. The relationship between internet use and health service utilization is complex and influenced by multiple factors [13,14,21]. Existing empirical studies [21-23] on the effect of internet health information on demand for health care services have shown unclear and inconsistent findings. Some studies [21,22] found that access to online health information enhanced the demand and use of health services by individuals. An empirical analysis based on data from the United States Health Information Trends Survey revealed that OHIS has a positive, relatively large, and statistically significant effect on individual health care demand [21]. Another cross-sectional study [22] of 993 adults in Vietnam drew positive conclusions that the prevalent use of mass media sources, including the internet, for health information was associated with higher rates of health care utilization, even though there were issues such as neglecting the quality of health information. However, a study [23] also found that the behavior of searching for online health information can negatively impact the utilization of health services. A Chinese nationally representative data analysis based on an instrumental variable regression revealed that mobile internet use significantly diminished the inclination of an individual to seek primary health care services [23].

Seeking information on the internet can enhance the knowledge of individuals about health concerns and symptoms, enabling them to better manage their health conditions [24]. However, a vast volume of health information needs to be screened and evaluated, and the perception of people toward health information may influence this process. On the one hand, OHIS fulfills the need of patients for health information or provides opportunities to interact with health providers through the internet, which may decrease the frequency of health consultation visits. In contrast, OHIS draws the attention of patients to health issues, increasing their demand and frequency of visits to health services. Currently, a substantial body of theoretical work has delved into the factors influencing OHIS behavior [25]. Particular emphasis has been placed on the mechanisms by which the attitudes and perspectives of individuals regarding online health information shape their corresponding behaviors [25]. A systematic review integrated the comprehensive model of information seeking, the planned risk information seeking model, and the situational theory of problem-solving and found that the perception and trust of people in online information were the most dominant predictors of OHIS behavior [25]. For patients with chronic conditions, perceived susceptibility and severity can effectively explain perceived risk, helping predict their OHIS behavior. Informational and emotional support can help patients perceive benefits, thereby positively affecting their OHIS behavior [26]. Simultaneously, the OHIS attitude and behavior are dependent on the identity of those who offer opinions online; thus, the advice obtained from others with similar interests is given more weight [27]. A study involving 10,000 participants from mainland China found that perceived information quality mediates the influence of OHIS on health behavior [28].

The abovementioned studies have demonstrated that the impact of OHIS behavior on health service utilization requires considering attitudes, perspectives, and views of people on online health information, which has been confirmed as an important factor for the acceptance of health information [29]. How people perceive the health information available online influences subsequent processes on health behaviors or the use of health services, for example, if they believe that information on the internet has a positive impact on their health, whether the online information helps them understand the doctor’s advice, or whether the doctor provided appropriate advice. Although existing literature [13-19,21-23] has demonstrated OHIS prevalence among different populations and patients as well as the factors that influence OHIS, there has been minimal discussion on the associations between OHIS and health services. Furthermore, even fewer studies have explored the impact of this association [25]. More research is needed regarding the associations among people’s attitudes, perceptions, OHIS, and health services.

The above discussion suggests that a step toward a comprehensive understanding of the effects described requires the development of a complex model that considers the simultaneous relationships among OHIS, health service utilization, and the attitude and perceptions of an individual. The research questions of this study focus on how OHIS behavior affects the practice of health service utilization and how the attitudes and perceptions of people toward online health information play a role in this process. We developed a joint model of OHIS, health service usage, and health information belief that considers an individual’s unobserved characteristics that are likely to be correlated with health information seeking behavior, individual’s health status, and health care utilization.

## Methods

### Study Participants

This study analyzes data from the Chinese General Social Survey (CGSS), which systematically monitors the changing relationship between the social structure and the quality of life in both urban and rural China. The CGSS is China’s first, nationally representative and comprehensive continuous academic survey initiative conducted by an academic institution in mainland China [30]. This project collects data by using a stratified, multistage probability proportional to size method. Data are comprehensively gathered at multiple levels, including society, communities, households, and individuals. CGSS covers 28 provinces, autonomous regions, and municipalities across China. In this study, the latest CGSS2021 survey was selected as the data source for the analysis, which included the context on OHIS and the perceptions of respondents. The details of the sampling scheme and design are available on the official CGSS website [30].

**Figure 1:**
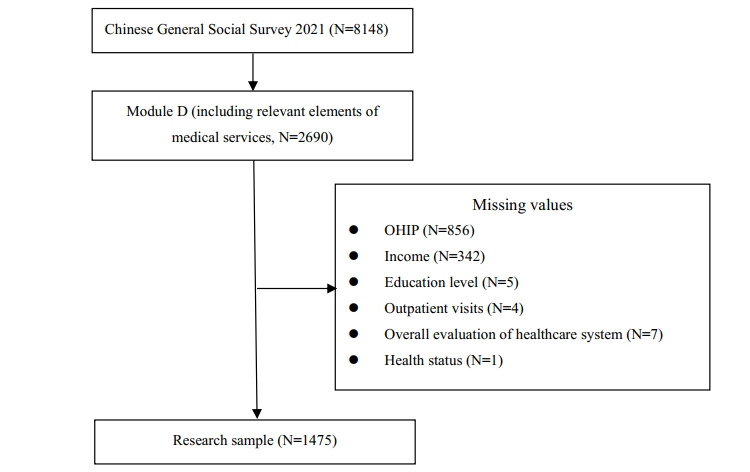
Participant screening flowchart. OHIP: online health information perception.

A total of 8148 samples were included in the CGSS2021 data, with 2690 participating in the medical service–related questions (module D). After removing the missing values for the key variables such as online health information perception (OHIP) (n=856), income (n=342), education level (n=5), outpatient visits (n=4), overall evaluation of the health care system (n=7), and health status (n=1), 1475 study samples were obtained. The specific data processing procedures are represented in Figure 1.

### Ethics Approval

CGSS was approved by the ethics review committee of Renmin University of China. The study data were anonymous, and the answers were protected by privacy law. The data collection and storage protocols were in full compliance with the personal information protection law of China. All obtained data were anonymized, which means removing any identifiers that can directly or indirectly link the data to individual participants. Each participant signed an informed consent form at the time of participation. There was no requirement for additional ethics approval for approved data users.

### Measures

#### Outcome Variable

The health service utilization by respondents served as the outcome variable for this analysis. This variable was represented by the question “In the last 12 months, how often have you visited a doctor?” The questionnaire was divided into 2 parts: visits to traditional Chinese medicine (TCM) physicians and visits to Western medicine physicians. The numbers 1, 2, 3, 4, and 5 were assigned to the answers never, rarely, sometimes, often, and very often, respectively. The 2 answers were combined to create the outpatient utilization frequency variable ranging from 2 to 10. OHIS behavior was selected as an independent variable in our analysis. OHIS was described by the question, “In the past 12 months, how often have you searched for health or medical information for yourself or others online in various forms?” OHIS was identified as yes if respondents selected several times a day, once a day, or a few times a week/month/year. Other responses (never go online, hardly ever, and cannot choose) were assigned to “no” in the analysis. Afterward, further analysis was conducted after removing individuals with an answer “no” on the OHIS behavior. For those with OHIS behavior, the numbers 1, 2, 3, 4, and 5 were assigned to the answers a few times a year, a few times a month, a few times a week, once a day, and several times a day, respectively.

#### Mediating Variables

The perceptions of respondents regarding online health information were selected as the mediating variables in this analysis. In the CGSS2021 questionnaire, 4 questions were selected to represent perception: “Do you agree with the following statement that information on the internet has positively influenced my health behaviors?”, “Do you agree with the following statement that information on the internet has helped me understand what my doctor has told me?”, “Do you agree with the following statement that the internet can help people decide if they need to go to the doctor or not?”, and “Do you agree with the following statement that the internet can help to make sure that doctors are giving people the right advice?” The numbers 1, 2, 3, 4, and 5 were assigned to the responses strongly disagree, disagree, cannot tell, agree, and strongly agree, respectively. Factor analysis was conducted to extract the perception of respondents for online information from the 4 questions listed above. The factor with a contribution rate of 0.548 was extracted according to the criterion that the eigen value is greater than 1. The procedure and results of factor analysis are given in Multimedia Appendix 1.

#### Control Variables

The control variables selected for this study were as follows: gender (female=0, male=1), age, income, residence (rural=0, urban=1), marital status (unmarried=0, married=1), education level (primary or below=0, junior middle school=1, high school or above=1), health status (very poor=0, relatively poor=1, general=2, relatively good=3, good=4), region (western region=0, central region=1, and eastern region=2), and their overall evaluation of the health care system (not at all satisfied=0, very dissatisfied=1, quiet dissatisfied=2, cannot say=3, quite satisfied=4, very satisfied=5, and fully satisfied=6).

### Statistical Analysis

Descriptive and univariate analyses were performed for the basic characteristics of respondents. A chi-square test was used for discrete variables, while *t* tests (2-tailed) were applied for normal or 2-sample data. Wilcoxon rank-sum tests were applied for nonnormal or unequal variance continuous variables. Additionally, ordinary least squares regression was conducted to examine the effect of OHIS on physician visits. Bootstrap methods were used to assess the mediation effects of health information perception; 95% CI and *P* values were presented. The weights provided by the CGSS data were utilized for regression models and mediation analysis in this study for ensuring generalizability and reliability. Statistical analysis was conducted using Stata (version 14.0) for Microsoft Windows (Stata Corp).

## Results

### Sample Characteristics

A total of 1475 cases were selected from the database for the analysis. The mean age of the respondents was 46.72 (SD 15.864) years, 794 (53.83%) were females, 1083 (73.42%) were married, and 562 (38.10%) were living in rural areas. A total of 420 respondents were western region residents, representing 28.47% (420/1475) of the total number of respondents, while 26.31% (388/1475) were central region residents, and 45.22% (667/1475) were eastern region residents. Almost half (728/1475, 49.36%) of the respondents had a high school diploma or more, while one-fifth (289/1475, 19.59%) had only completed primary school or were illiterate. More than half of the respondents (853/1475, 57.83%) were in relatively good or good health conditions. The frequency of physician visits in the last 12 months was 3.883 (SD 1.591); that of TCM visits was 1.787 (SD 0.884) and that of Western outpatient visits was 2.096 (SD 0.957). Among the respondents, 939 (63.66%) sought online health information in the last 12 months.

One-way analysis of the number of total clinic, TCM, and Western physician visits was conducted for the related variables. Preliminary analysis revealed differences among respondents with different health status, genders, and education levels. The female respondents visited physicians more frequently than male respondents in the last 12 months (*t*_1473_=–3.411; *P*=.007), as evidenced by the number of visits for TCM outpatients (*t*_1473_=3.040; *P*=.02) and Western medicine outpatients (*t*_1473_=2.842; *P*=.002). Those with primary school or less education had the highest number of physician visits (*F*_2,1472_=5.37; *P*=.04), TCM visits (*F*_2,1472_=6.46; *P*=.002), and Western clinic visits (*F*_2,1472_=3.09; *P*=.046). As the health condition worsened, the overall number of physician visits increased (*F*_4,1470_=52.33; *P*<.001), and the same trend of changes was observed for both TCM (*F*_4,1470_=55.73; *P*<.001) and Western clinic visits (*F*_4,1470_=24.03; *P*<.001). More details on the sample statistic descriptions are provided in Table 1.

**Table 1:** Characteristics of the participants in relation to physician visits (N=1475).

Indicators/variables			Values, n (%)		Physician visits									Traditional Chinese Medicine visits										Western clinic visits						
					Mean (SD)		*t* test *(df)/F* test *(df)*			*P* value			Mean (SD)			*t* test *(df)/F* test *(df)*			*P* value			Mean (SD)				*t* test *(df)/F* test *(df)*			*P* value	
**Gender^a^**								–3.411 (1473)			.007						3.040 (1473)			.002							2.842 (1473)			.002
	Female	794 (53.83)		4.01 (1.656)								2.166 (0.034)									1.847 (0.032)									
	Male	681 (46.17)		3.73 (1.499)								2.014 (0.035)									1.716 (0.031)									
**Education level^a^**								5.37 (2, 1472)			.04						6.46 (2, 1472)			.002							3.09 (2, 1472)			.046
	Primary and below	289 (19.59)		4.418 (1.734)								2.276 (1.037)									1.871 (0.946)									
	Junior school	458 (31.05)		3.770 (1.571)								2.058 (0.989)									1.711 (0.867)									
	High school and above	728 (49.36)		3.848 (1.534)								2.048 (0.894)									1.800 (0.866)									
**Online health information seeking behavior^a^**								–0.322 (1473)			.75						0.192 (1473)			.85							–0.789 (1473)			.43
	No	536 (36.34)		3.865 (1.745)								2.102 (0.045)									1.763 (0.040)									
	Yes	939 (63.66)		3.893 (1.497)								2.092 (0.029)									1.800 (0.027)									
**Residence^a^**								1.028 (1473)			.30						1.563 (1473)			.12							0.159 (1473)			.87
	Rural	562 (38.10)		3.937 (1.639)								2.145 (0.042)									1.791 (0.037)									
	Urban	913 (61.90)		3.849 (1.561)								2.065 (0.030)									1.784 (0.029)									
**Health status^b^**								52.33 (4, 1470)			<.001						55.73 (4, 1470)			<.001							24.03 (4, 1470)			<.001
	Very poor	45 (3.05)		5.666 (1.906)								3.044 (1.205)									2.622 (1.113)									
	Relatively poor	127 (8.61)		4.976 (1.738)								2.795 (1.078)									2.181 (1.072)									
	Average	450 (30.51)		4.133 (1.607)								2.275 (0.985)									1.857 (0.928)									
	Relative good	566 (38.37)		3.584 (1.377)								1.916 (0.816)									1.667 (0.761)									
	Good	287 (19.46)		3.317 (1.317)								1.710 (0.721)									1.606 (0.767)									
**Marital status^a^**								0.618 (1473)			.53						0.816 (1473)			.41							0.229 (1473)			.82
	Unmarried	392 (26.58)		3.926 (1.539)								2.130 (0.046)									1.795 (0.044)									
	Married	1083 (73.42)		3.867 (1.610)								2.084 (0.029)									1.783 (0.026)									
**Overall evaluation of the health care system^b^**								1.30 (6, 1468)			.25						0.98 (6, 1468)			.43							1.82 (6, 1468)			.09
	Not at all satisfied	5 (0.34)		3.400 (1.341								1.80 (1.095									1.60 (1.341									
	Very dissatisfied	22 (1.49)		3.454 (1.625								1.727 (0.935									1.727 (0.882									
	Quite dissatisfied	76 (5.15)		3.894 (1.536)								2.105 (0.903)									1.789 (0.942)									
	Can’t say	225 (15.25)		4.097 (1.586)								2.137 (0.922)									1.96 (0.927)									
	Quite satisfied	739 (50.10)		3.887 (1.547)								2.120 (0.944)									1.767 (0.845)									
	Very satisfied	280 (18.98)		3.771 (1.603)								2.032 (0.966)									1.739 (0.863)									
	Fully satisfied	128 (8.68)		3.812 (1.830)								2.093 (1.089)									1.718 (0.995)									
**Region^b^**								1.46 (2, 1472)			.23						0.04 (2, 1472)			.96							3.85 (2, 1472)			.02
	Western	420 (28.47)		3.99 (1.709)								2.107 (1.001)									1.888 (0.944)									
	Central	388 (26.31)		3.829 (1.529)								2.087 (0.910)									1.742 (0.841)									
	Eastern	667 (45.22)		3.844 (1.548)								2.094 (0.951)									1.749 (0.865)									
Total			1475		3.883 (1.591)		N/A^c^			N/A			1.787 (0.884)			N/A			N/A			2.096 (0.957)				N/A			N/A	

^a^*t* test.

^b^*F* test.

^c^N/A: not applicable.

### Multivariate Regression

After adjusting for other variables, those with OHIS behaviors increased the frequency of outpatient visits by 0.289 times more during the past year compared to those without OHIS behaviors (*P*=.003). They increased at a rate of 0.131 times in TCM outpatient visits (*P*=.01) and 0.158 times in Western physician visits (*P*=.007). Moreover, multiple regression analyses found statistically significant effects of gender, age, and health status on physician visits. Relative to the female respondents, males decreased their overall physician, TCM, and Western medicine visits by 23% (*P*=.006), 9.3% (*P*=.04), and 13.7% (*P*=.006), respectively. The number of physician visits increased with age. For every 1-year increase in age, the overall physician visits increased by 1.5% (*P*<.001), TCM visits by 0.87% (*P*<.001), and Western medicine visits by 0.7% (*P*<.001). Similar to the results of the univariate analyses, respondents with poorer health had more medical visits, while those in good health had significantly fewer visits to physicians, TCM physicians, and Western physicians (*P*<.001). All the abovementioned factors affected the number of visits in all 3 types. Concurrently, some factors such as marital status, education level, and region of residence influenced one or two types of physician visits. In comparison with respondents who were not married, those who were married had lesser number of overall physician visits by 20.4% (*P*=.03), Western medicine visits by 15.5% (*P*=.008), and TCM visits by 4.8%, with a statistically nonsignificant difference (*P*=.36). In contrast to the respondents in western regions, those in the central and eastern regions had fewer TCM visits by 12.2% (*P*=.06) and 12.6% (*P*=.03), respectively. More details of the multiple regressions are given in Table 2.

**Table 2:** Multivariate associations between participant characteristics and physician visits (N=1475).

			Total physician visits							Traditional Chinese medicine visits							Western medicine physician visits				
			Coefficient (SE)		95% CI		*P* value		Coefficient (SE)			95% CI		*P* value		Coefficient (SE)			95% CI		*P* value
**Online health information seeking behavior**																					
	No	Ref^a^		Ref		Ref		Ref			Ref		Ref		Ref			Ref		Ref	
	Yes	0.289 (0.096)		0.100 to 0.478		.003		0.131 (0.052)			0.029 to 0.234		.01		0.158 (0.058)			0.044 to 0.271		.007	
**Gender**																					
	Female	Ref		Ref		Ref		Ref			Ref		Ref		Ref			Ref		Ref	
	Male	–0.230 (0.083)		–0.393 to –0.067		.006		–0.093 (0.045)			–0.184 to –0.002		.04		–0.137 (0.050)			–0.233 to –0.039		.006	
Age			0.015 (0.003)		0.008 to 0.022		<.001		0.007 (0.001)			0.004 to 0.011		<.001		0.007 (0.002)			0.002 to 0.011		.001
**Education**																					
	Primary or below	Ref		Ref		Ref		Ref			Ref		Ref		Ref			Ref		Ref	
	Junior middle school	–0.021 (0.133)		–0.283 to 0.239		.87		0.012 (0.070)			–0.126 to 0.151		.86		–0.034 (0.083)			–0.196 to 0.127		.68	
	High school or above	0.256 (0.140)		–0.019 to 0.533		.07		0.162 (0.073)			0.018 to 0.306		.03		0.094 (0.087)			–0.076 to 0.264		.28	
Income			–0.003 (0.018)		–0.040 to 0.032		.83		0.004 (0.009)			–0.015 to 0.023		.66		–0.008 (0.012)			–0.030 to 0.014		.47
**Marital status**																					
	Unmarried	Ref		Ref		Ref		Ref			Ref		Ref		Ref			Ref		Ref	
	Married	–0.204 (0.095)		–0.391 to –0.016		.03		–0.048 (0.053)			–0.154 to 0.056		.36		–0.155 (0.058)			–0.269 to –0.041		.008	
**Residence**																					
	Rural	Ref		Ref		Ref		Ref			Ref		Ref		Ref			Ref		Ref	
	Urban	–0.019 (0.091)		–0.200 to 0.160		.83		0.010 (0.051)			–0.090 to 0.110		.84		–0.030 (0.055)			–0.137 to 0.077		.58	
**Region**																					
	Western region	Ref		Ref		Ref		Ref			Ref		Ref		Ref			Ref		Ref	
	Central region	–0.085 (0.116)		–0.313 to 0.142		.46		–0.122 (0.063)			–0.247 to 0.002		.06		0.037 (0.068)			–0.097 to 0.170		.59	
	Eastern region	–0.066 (0.104)		–0.271 to 0.138		.53		–0.126 (0.059)			–0.241 to –0.010		.03		0.060 (0.062)			–0.061 to 0.180		.34	
**Health status**																					
	Very poor	Ref		Ref		Ref		Ref			Ref		Ref		Ref			Ref		Ref	
	Relatively poor	–0.811 (0.353)		–1.504 to –0.117		.02		–0.512 (0.206)			–0.917 to –0.107		.01		–0.298 (0.219)			–0.727 to 0.130		.17	
	General health	–1.605 (0.335)		–2.263 to –0.947		<.001		–0.884 (0.193)			–1.262 to –0.505		<.001		–0.721 (0.205)			–1.122 to –0.320		<.001	
	Relatively good	–2.001 (0.336)		–2.662 to –1.341		<.001		–0.977 (0.193)			–1.356 to –0.598		<.001		–1.024 (0.204)			–1.425 to –0.622		<.001	
	Good health	–2.274 (0.344)		–2.949 to –1.599		<.001		–1.040 (0.197)			–1.427 to –0.654		<.001		–1.234 (0.208)			–1.641 to –0.826		<.001	
**Overall evaluation of the health care system**																					
	Not at all satisfied	Ref		Ref		Ref		Ref			Ref		Ref		Ref			Ref		Ref	
	Very dissatisfied	0.831 (0.483)		–0.117 to 1.779		.09		0.449 (0.396)			–0.328 to 1.229		.26		0.382 (0.295)			–0.196 to 0.961		.19	
	Quiet dissatisfied	0.841 (0.419)		0.019 to 1.663		.045		0.211 (0.363)			–0.500 to 0.923		.56		0.630 (0.267)			0.105 to 1.154		.02	
	Can’t say	1.183 (0.398)		0.402 to 1.965		.003		0.485 (0.355)			–0.211 to 1.182		.17		0.698 (0.253)			0.202 to 1.194		.006	
	Quite satisfied	1.116 (0.390)		0.351 to 1.814		.004		0.342 (0.350)			–0.345 to 1.031		.33		0.773 (0.250)			0.283 to 1.263		.002	
	Very satisfied	1.010 (0.394)		0.236 to 1.784		.01		0.327 (0.353)			–0.365 to 1.019		.35		0.683 (0.252)			0.189 to 1.177		.007	
	Fully satisfied	0.835 (0.415)		0.021 to 1.649		.04		0.235 (0.360)			–0.472 to 0.942		.51		0.600 (0.262)			0.086 to 1.113		.02	
Constant variable			3.920 (0.531)		2.877 to 4.962		<.001		1.904 (0.390)			1.138 to 2.670		<.001		2.015 (0.330)			1.366 to 2.664		<.001

^a^Ref: reference value.

Finally, the group that never participated in OHIS was removed. Our findings indicate that a higher frequency of OHIS was associated with a higher frequency of outpatient visits (β=0.127; *P*=.003) and TCM visits (β=0.082; *P*=.001). More details are given in Table 3.

**Table 3:** Multivariate associations between participant characteristics and physician visits among those with online health information seeking behaviors (n=939).

			Total physician visits							Traditional Chinese medicine visits							Western medicine physician visits				
			Coefficient (SE)		95% CI		*P* value		Coefficient (SE)			95% CI		*P* value		Coefficient (SE)			95% CI		*P* value
Online health information seeking frequency			0.127 (0.043)		0.042 to 0.211		.003		0.082 (0.025)			0.033 to 0.131		.001		0.045 (0.028)			–0.010 to 0.099		.11
**Gender**																					
	Female	Ref^a^		Ref		Ref		Ref			Ref		Ref		Ref			Ref		Ref	
	Male	–0.133 (0.102)		–0.334 to 0.067		.19		–0.044 (0.057)			–0.156 to 0.068		.44		–0.089 (0.061)			–0.209 to 0.031		.15	
Age			0.008 (0.004)		0.000 to 0.017		.046		0.006 (0.002)			0.001 to 0.011		.01		0.002 (0.002)			–0.003 to 0.008		.34
**Education**																					
	Primary or below	Ref		Ref		Ref		Ref			Ref to		Ref		Ref			Ref		Ref	
	Junior middle school	–0.215 (0.167)		–0.543 to 0.113		.20		–0.037 (0.095)			–0.223 to 0.149		.69		–0.178 (0.105)			–0.384 to 0.029		.09	
	High school or above	0.145 (0.167)		–0.182 to 0.472		.39		0.152 (0.094)			–0.032 to 0.336		.11		–0.007 (0.104)			–0.211 to 0.197		.95	
Income			0.005 (0.021)		–0.037 to 0.047		.82		.004 (0.011)			–0.017 to 0.026		.69		0.001 (0.014)			–0.027 to 0.028		.97
**Marital status**																					
	Unmarried	Ref		Ref		Ref		Ref			Ref		Ref		Ref			Ref		Ref	
	Married	–0.132 (0.115)		–0.357 to 0.093		.25		–0.021 (0.064)			–0.146 to 0.104		.74		–0.111 (0.072)			–0.252 to 0.030		.12	
**Residence**																					
	Rural	Ref		Ref		Ref		Ref			Ref		Ref		Ref			Ref		Ref	
	Urban	0.021 (0.116)		–0.207 to 0.249		.86		–0.004 (0.065)			–0.132 to 0.124		.95		0.025 (0.069)			–0.109 to 0.160		.72	
**Region**																					
	Western region	Ref		Ref		Ref		Ref			Ref		Ref		Ref			Ref		Ref	
	Central region	–0.131 (0.141)		–0.407 to 0.146		.35		–0.144 (0.080)			–0.300 to 0.012		.07		0.014 (0.082)			–0.148 to 0.175		.87	
	Eastern region	–0.114 (0.131)		–0.371 to 0.144		.39		–0.144 (0.074)			–0.289 to 0.001		.05		0.030 (0.080)			–0.127 to 0.188		.71	
**Health status**																					
	Very poor	Ref		Ref		Ref		Ref			Ref		Ref		Ref			Ref		Ref	
	Relatively poor	–0.573 (0.513)		–1.579 to 0.434		.27		–0.520 (0.311)			–1.131 to 0.091		.09		–0.053 (0.314)			–0.669 to 0.563		.87	
	General health	–1.253 (0.493)		–2.221 to –0.285		.01		–0.871 (0.294)			–1.448 to –0.293		.003		–0.382 (0.290)			–0.951 to 0.188		.19	
	Relatively good	–1.846 (0.490)		–2.806 to –0.885		<.001		–1.047 (0.293)			–1.622 to –0.472		<.001		–0.798 (0.287)			–1.362 to –0.235		.006	
	Good health	–1.968 (0.501)		–2.951 to –0.985		<.001		–1.039 (0.299)			–1.626 to –0.453		.001		–0.929 (0.292)			–1.502 to –0.356		.002	
**Overall evaluation of the health care system**																					
	Not at all satisfied	Ref		Ref		Ref		Ref			Ref		Ref		Ref			Ref		Ref	
	Very dissatisfied	1.461 (0.691)		0.105 to 2.817		.04		0.815 (0.230)			0.363 to 1.267		<.001		0.646 (0.600)			–0.356 to 1.823		.28	
	Quiet dissatisfied	1.620 (0.596)		0.449 to 2.790		.007		0.771 (0.147)			0.483 to 1.060		<.001		0.848 (0.570)			–0.271 to 1.967		.14	
	Can’t say	1.914 (0.572)		0.791 to 3.037		.001		1.032 (0.118)			0.800 to 1.265		<.001		0.882 (0.558)			–0.213 to 1.976		.11	
	Quite satisfied	1.862 (0.564)		0.756 to 2.968		.001		0.903 (0.100)			0.707 to 1.099		<.001		0.959 (0.556)			–0.133 to 2.050		.08	
	Very satisfied	1.726 (0.570)		0.606 to 2.845		.003		0.872 (0.108)			0.660 to 1.083		<.001		0.854			–0.241 to 1.949		.13	
	Fully satisfied	1.526 (0.600)		0.350 to 2.703		.01		0.751 (0.154)			0.449 to 1.053		<.001		0.776 (0.568)			–0.340 to 1.891		.17	

^a^Ref: reference value.

### Mediation Effect Analysis

**Figure 2:**
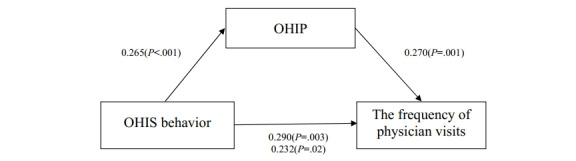
The mediating role of online health information perception between online health information seeking behavior and physician visits. OHIP: online health information perception; OHIS: online health information seeking.

First, the total effect of OHIS behavior on the frequency of physician visits was examined. The results revealed that OHIS had a significant influence on total physician visits (β=0.290; *P=*.003), indicating a certain correlation between OHIS behavior and physician visits. We also tested the effect of OHIS behavior on perception, and results revealed that OHIS had a significant positive impact on perception (β=0.265; *P<*.001). Furthermore, the effect of OHIP on the number of physician visits was conducted, and it was found to have a significant effect on physician visits (β=0.270; *P*=.001). Finally, the effect of perception on physician visits while controlling for OHIS behavior was also assessed. We found that the perception of people regarding online health information had a significantly positive influence on physician visits (β=0.232; *P=*.02). More details are given in Table 4 and Figure 2.

**Table 4:** Mediation effect analysis of online health information seeking behavior, physician visits, and online health information perception (N=1475).

		Model 1 (N=1475)			Model 2 (N=1475)			Model 3 (N=1475)			Model 4 (N=1475)	
		Physician visits	*P* value	Online health information perception		*P* value	Physician visits		*P* value	Physician visits		*P* value
Online health information seeking behavior		0.290 (0.096)	.003	0.265 (0.032)		<.001	N/A^a^		N/A	0.232 (0.100)		.01
Online health information perception		N/A	N/A	N/A		N/A	0.270 (0.082)		.001	0.217 (0.084)		.02
Gender		–0.230 (0.083)	.006	–0.000 (0.028)		.99	–0.242 (0.083)		.004	–0.230 (0.083)		.006
Residence		–0.020 (0.092)	.83	–0.028 (0.030)		.35	0.000 (0.091)		.99	–0.014 (0.092)		.88
Age		0.015 (0.004)	<.001	0.000 (0.001)		.82	0.013 (0.004)		<.001	0.015 (0.004)		<.001
**Education levels**												
	Primary and low	Reference		Reference			Reference			Reference		
	Junior high school	–0.022 (0.133)	.87	0.010 (0.041)		.81	–0.005 (0.133)		.97	–0.024 (0.133)		.86
	High school and above	0.257 (0.141)	.07	0.011 (0.043)		.80	0.297^**^ (0.141)		.04	0.254 (0.140)		.07
Income		–0.004 (0.019)	.83	0.009 (0.008)		.24	–0.004 (0.019)		.83	–0.006 (0.019)		.75
**Health condition**												
	Very poor	Reference		Reference			Reference			Reference		
	Relatively poor	–0.811 (0.353)	<.001	–0.063 (0.107)		.55	–0.807 (0.354)		.02	–0.797 (0.355)		.03
	Average health	–1.605 (0.336)	<.001	–0.051 (0.095)		.59	–1.601 (0.336)		<.001	–1.594 (0.338)		<.001
	Relatively good	–2.002 (0.337)	<.001	–0.077 (0.096)		.43	–1.985 (0.337)		<.001	–1.985 (0.339)		<.001
	Good health	–2.275 (0.344)	<.001	–0.038 (0.099)		.70	–2.285 (0.345)		<.001	–2.266 (0.347)		<.001
**Marital status**												
	Unmarried	Reference		Reference			Reference			Reference		
	Married	–0.204 (0.096)	.03	–0.013 (0.032)		.68	–0.186 (0.094)		.048	–0.202 (0.095)		.03
**Overall evaluation of the health care system**												
	Not at all satisfied	Reference		Reference			Reference			Reference		
	Very dissatisfied	0.831 (0.483)	.09	–0.081 (0.281)		.77	0.915 (0.495)		.06	0.849 (0.511)		.09
	Quiet dissatisfied	0.841 (0.419)	.045	0.204 (0.223)		.36	0.844 (0.425)		.047	0.797 (0.444)		.07
	Can’t say	1.184 (0.399)	.003	0.132 (0.218)		.55	1.206 (0.405)		.003	1.155 (0.424)		.007
	Quite satisfied	1.116 (0.390)	.004	0.181 (0.217)		.40	1.115 (0.398)		.005	1.077 (0.417)		.01
	Very satisfied	1.011 (0.395)	.01	0.244 (0.217)		.26	0.988 (0.402)		.01	0.958 (0.421)		.02
	Fully satisfied	0.835 (0.415)	.04	0.352 (0.222)		.11	0.781 (0.423)		.07	0.759 (0.441)		.09
**Region**												
	Western	Reference		Reference			Reference			Reference		
	Central	–0.086 (0.116)	.46	0.118 (0.037)		.001	–0.111 (0.116)		.34	–0.112 (0.116)		.34
	Eastern	–0.067 (0.105)	.53	–0.005 (0.035)		.89	–0.065 (0.104)		.53	–0.066 (0.104)		.53

^a^N/A: not applicable.

## Discussion

### Primary Findings

The key findings of this study are summarized as follows. First, the perception of people regarding online health information influences the effect of OHIS on health service usage as predicted. The increased prevalence of OHIS behaviors in the internet era necessitates more awareness and discrimination of health information accessed on the internet. Our findings validated the impact of OHIS on health service utilization outcomes. Second, the effect found through the mediation analysis was partial mediating, which can be divided into 2 parts. On the one hand, respondents’ OHIS behavior directly affects the number of physician visits. On the other hand, OHIS behavior affects their perception of the health information available online, which subsequently affects their use of outpatient services. Third, the multivariable linear regression results revealed that OHIS behavior significantly increased the utilization of clinic visits after controlling for other variables. Those with OHIS behavior showed 0.308 times increase in the frequency of total physician visits compared to those without OHIS behaviors. This trend was not only reflected in the total number of physician visits but also in the TCM and Western medicine physician visits. Finally, based on the joint model that was built on OHIS, health care utilization, and OHIP, we clarified the mediator roles of OHIP between OHIS behavior and physician visits and provided new perceptive and explanatory mechanisms to explore the relationship between health information seeking behavior and health service utilization.

### Interpretation and Comparison With Existing Literature

Studies exploring the determinants of OHIS have confirmed that perceived usefulness and attitude have a strong positive effect on OHIS [29]. A previous study [31] investigated the psychological mechanisms between OHIS behavior and doctor-patient interactions, concluding the mediating role of eHealth literacy, perceived disease severity, and action benefits. The findings of this study further confirm that a higher perception of health-related information online affects the OHIS behavior and influences the health utilization process. As various studies [14,32] have indicated, OHIS behavior can be a potential tool to improve health literacy improvement and health-related behaviors. Residents consciously accessing health information on the internet can be more knowledgeable about their health and are more likely to identify potential health problems, resulting in higher health demand and higher physician visits.

Based on previous studies, education level is one of the most important factors for health service utilization [33,34]. However, the impact of education level on outpatient services is complex. From one point of view, due to their strong health awareness, people with higher education levels may visit outpatient clinics more frequently for minor discomforts or for the management of chronic diseases. In contrast, individuals with a higher education level are inclined to possess more comprehensive preventive health care knowledge, higher health literacy, and maintain a superior state of physical well-being, which may also lead to a decrease in outpatient visits. We identified significant differences between different education levels and outpatient visits through a primary univariate analysis. Participants with primary or lower education levels had more physician visits. However, this trend disappeared in the multivariate analysis after controlling for other variables. Among educational attainment factors, having a high school diploma or above increased the likelihood of receiving TCM treatment, but this was not reflected in the overall and Western medicine treatments. In our analysis, increasing age indicated increased health service utilization, which is consistent with that reported in previous studies [21,23]. Older adults have various health needs, including medical checkups for chronic diseases, rehabilitation services after illness or surgery, mental health support to combat loneliness and depression, and long-term care assistance with daily activities. All these needs can be partly satisfied through the internet, which provides access to medical information, online consultations, health management, and virtual communities for support and interaction. However, 1 systematic review [7] indicated that older adults confronted 3 types of barriers to conducting OHIS: individual, social, and information and communication technology–related. The findings of this study indicated that shifting the perspective on OHIS can promote OHIS behavior and consequently enhance the rational use of health services. The older adults, being a vulnerable population in terms of health and information and communication technology, require additional support in using web-based services efficiently and reasonably.

Additionally, unauthenticated health information is widely available on the internet. Another study [35] revealed that 96% of the residents use unauthenticated health information to answer some health-related questions. Patients may not always be able to use effective online search strategies. Studies have indicated that when there is a contradiction between the information on the internet and that provided by doctors, people are more likely to trust the perspective of doctors [36,37]. These results verified that people’s positive perceptions and attitudes toward online health information promote OHIS behavior and the use of health services. Health care professionals’ recommendations and assistance on reliable information sources and trustworthy websites in patients’ search for online health information can be very useful and can improve the rationality of online health information and the seeking of medical treatment.

### Implications for Policies and Practice

The findings of this study have important implications for future policies and practices in the health care system. Focusing on the access of individuals to online health information allows health service providers to have a better understanding of their health knowledge, behaviors, and even health attitudes, promoting a more logical use of health services. In the field of health education, the primary health care service system aims to improve the eHealth literacy of residents, including their ability to seek, discriminate, judge, and use internet health information. This is done to promote their ability to access their own or their family’s health status and enhance their ability to make timely and appropriate health care decisions. The older adults, who are always considered medically underserved populations, often reported a lack of social support in their OHIS, including informational, organizational, instrumental, intergenerational, and peer support. Interventions can be explored to improve reasonable outpatient utilization by the older adults by improving their perception and attitude toward health information online. Comprehensive support, which encompasses user-friendly devices and software, training, and guidance on efficient internet use, accessible internet connections, and dedicated support services, is necessary for older adults to truly benefit from OHIS and reasonable health services.

### Strengths and Limitations

Compared with previous studies, this study is innovative and contributes in the following aspects. First, the research sample included is from CGSS, a large-scale, comprehensive social survey project. On this basis, people’s perception of online health information was selected as the mediating factor to explain the mechanism linking OHIS to health service utilization, broadening the research scope. Furthermore, these findings provide new evidence on OHIS behavior and physician visits and verified the partial mediation role of people’s perceptions and attitudes toward online health information. Lastly, multiple variables were integrated into participants’ perception of online health information based on the factor analysis, which enhanced its efficiency, rationality, and reliability.

Although this study achieved some useful results, there are some limitations. First, our study was conducted using cross-sectional data, which prevented us from determining the causal relationship between variables. Future studies can adopt a longitudinal design to further understand the dynamic relationships among OHIS, OHIP, and health services. Given that a public database was used as a resource, certain limitations were unavoidable. OHIP, selected as the mediating variable, was relatively subjective when compared with eHealth literacy or ability. Nevertheless, it remains significant to know that the attitudes of residents toward online health information have influenced their OHIS and health service utilization.

In the future, the OHIP mechanism can be further explored in more depth, and the influence can be expanded to inpatient and emergency service utilization. Then, the relationships among OHIS, OHIP, and health service utilization can be investigated in different contexts to improve the generalizability of the results. Finally, other research methods such as experimental research and case studies can be combined to gain a more comprehensive understanding of the relationships between variables.

### Conclusions

The OHIP of residents influences the effect of OHIS on the frequency of physician visits. OHIS behavior impacts outpatient service utilization both directly and indirectly via OHIP. OHIS behaviors significantly increase the frequency of physician visits after controlling for other variables. Interventions can be explored to improve the health utilization of residents by improving their OHIP.

## Appendix

Multimedia Appendix 1 Procedure and results of factor analysis.

## Abbreviations

CGSS: Chinese General Social Survey
OHIP: online health information perception
OHIS: online health information seeking
TCM: traditional Chinese medicine

## Data Availability

These data were derived from the Chinese General Social Survey, which is open to everyone. Researchers who want to use these data can visit the website [30]. The datasets generated and analyzed during this study are available from the corresponding author on reasonable request.
