# Supplementary material for: Associations Among Online Health Information Seeking Behavior, Online Health Information Perception, and Health Service Utilization: Cross-Sectional Study
Source: J Med Internet Res. 2025 Mar 14;27:e66683. doi: 10.2196/66683 (PMC11953594; doi:10.2196/66683)
Supplement: Multimedia Appendix 1 [file jmir_v27i1e66683_app1.docx]

APPENDIX 1

Table S1. Results of factor analysis

|  | **MEAN** | **S.D.** | **Factor explanatory power** | **Contribution rate** |
| --- | --- | --- | --- | --- |
| **Question 1** | 3.261 | 0.925 | 0.320 | 0.548 |
| **Question 2** | 3.460 | 0.867 | 0.354 |  |
| **Question 3** | 3.224 | 0.941 | 0.339 |  |
| **Question 4** | 3.390 | 0.851 | 0.337 |  |

Details of the questions:

1. Do you agree with the following statement that **Information on the internet has positively influenced my health behaviors**.

2. Do you agree with the following statement that **Information on the Internet has helped me understand what my doctor has told me**.

3. Do you agree with the following statement that **the internet can help people decide if they need to go to the doctor or not**.

4. Do you agree with the following statement that **the internet can help to make sure that doctors are giving people the right advice**.
